# Addressing gaps in surveillance: Use of emergency department accessibility and social vulnerability to identify potential wastewater surveillance sites in North Carolina

**DOI:** 10.1371/journal.pone.0346668

**Published:** 2026-08-17

**Authors:** Neha Shanker, Stacie Reckling, Nicole L. Snyder, Dianne Enright, Virginia T. Guidry, Ariel Christensen

**Affiliations:** 1 North Carolina Department of Health and Human Services, Division of Public Health, Raleigh, North Carolina, United States of America; 2 Center for Geospatial Analytics, North Carolina State University, Raleigh, North Carolina, United States of America; Retired-United States Environmental Protection Agency, UNITED STATES OF AMERICA

## Abstract

**Background:**

The North Carolina Division of Public Health relies on multiple complementary surveillance systems, including emergency department (ED) and wastewater surveillance, to track disease trends across the state. Despite having comprehensive statewide ED coverage, several areas have limited access to ED facilities and concurrent higher social vulnerability. In this analysis, we demonstrate complementarity between North Carolina ED and wastewater surveillance and assess geographic gaps in coverage to identify potential new wastewater monitoring sites in low-coverage, high social vulnerability areas.

**Methods:**

Weekly wastewater SARS-CoV-2 viral concentrations and percentage of COVID-like Illness (CLI) ED visits across current wastewater sites geographically overlapping with hospitals, were compared using a Spearman’s rank-order correlation analysis for the period of November 2021 to August 2024. A drive-time analysis was conducted for 133 hospitals to identify areas within a 20–60-minute commute time to any ED facility. Out of a statewide sewershed polygon dataset, sewersheds serving a population greater than 1200 (n = 220) were selected for further investigation. ED service areas were then examined for overlap with sewersheds, and Social Vulnerability Index (SVI) rankings were compared for sites with a majority geographic overlap (>=50%).

**Results:**

Of the 28 overlapping hospitals, 26 show a moderate to strong correlation (rho = 0.303–0.718) with their respective sewersheds, demonstrating complementarity between the two systems. Of the 220 potential sewersheds examined, 40 overlapped with the 20–60-minute drive times to EDs. Of these, 26 wastewater sites had an overall SVI greater than 0.5 and were identified as priority additions to North Carolina’s wastewater surveillance network, including 13 with an overall SVI greater than 0.75.

**Conclusion:**

We used a novel geospatial approach to identify 26 priority wastewater sites with moderate to high social vulnerability and low ED accessibility. Additionally, this work further supports complementarity between ED and wastewater surveillance systems.

## Introduction

The North Carolina Department of Health and Human Services (NC DHHS) relies on multiple surveillance systems, including clinical and wastewater, to track disease trends across the state. Clinical surveillance in North Carolina is conducted in part by the collection and analysis of emergency department (ED) data from 132 hospitals across the state. The state’s in-house syndromic surveillance system, North Carolina Disease Event Tracking and Epidemiologic Collection Tool (NC DETECT) [1], collects near-real-time data on ED visits for several communicable and non-communicable health outcomes. To monitor specific illnesses, ED visits are classified into syndromes based on a keyword and/or diagnosis code-based search within the chief complaint, triage note, and diagnosis code fields. The ED visits observed for specific illnesses and aggregated trends are then monitored to detect aberrations and evaluate and respond to disease outbreaks or environmental health hazards. For example, COVID-like Illness (CLI) is a metric used to identify COVID-19 associated ED visits, and is often utilized as a substitute measure for confirmed case trends, particularly after the federal cessation of the COVID-19 public health emergency [2]. The National Syndromic Surveillance Program (NSSP) tracking [3] and NC DETECT iteratively utilize keyword and diagnosis code based queries such as CLI to search within hospital ED visits for symptoms and diagnoses of COVID-19.

While clinical surveillance has historically been used to track and monitor diseases like COVID-19 in North Carolina, wastewater surveillance emerged as a complementary and cost-effective tool for monitoring the presence and spread of SARS-CoV-2 during the COVID-19 pandemic. Wastewater surveillance measures viral nucleic acids in a sample of untreated wastewater and can capture health information about people living or working in the sewershed, or the community area upstream of a sampling site. An added benefit of wastewater surveillance is that it includes information about asymptomatic infections and cases that are not reported to a health care provider. In addition, prior studies have demonstrated strong complementarity between wastewater surveillance and clinical case trends in the United States [4–14], and globally [15–21], including correlations between wastewater viral concentrations and COVID-19-associated ED visit percentages [22]. For this reason, as well as others, wastewater surveillance data is increasingly being used by state and local health departments alongside case-based and syndromic surveillance data to inform public health decision-making.

North Carolina’s wastewater surveillance program, the North Carolina Wastewater Monitoring Network (NCWMN), began in 2021 with 28 sampling sites and expanded to a maximum of 50 sites in 2023. The NCWMN routinely analyzes wastewater from 34 sites for COVID-19, influenza A, influenza B, and respiratory syncytial virus, and maintains the flexibility to rapidly pivot to additional priority pathogens as emerging public health needs arise. Several studies over the past five years have assessed the relationship between COVID-19 trends between wastewater surveillance and clinical data in North Carolina demonstrating complementarity between the two systems [23,24]. Given that many of the NCWMN’s monitoring sites contain hospitals reporting to NC DETECT, a direct comparison of CLI visits at EDs with wastewater viral concentrations in the corresponding sewersheds could further strengthen evidence of complementarity.

Although North Carolina has a robust system of EDs across the state that collect clinical surveillance data, health information for people with limited access to ED facilities can potentially be missed from the state’s surveillance data. Thus, adding wastewater monitoring sites in areas with limited ED access can fill in the gaps in clinical surveillance thereby expanding coverage. Furthermore, areas with lower access to EDs often include rural populations that are more likely to experience disparities in health outcomes due to underlying issues like increased rates of poverty or lack of health insurance [25]. The CDC’s Social Vulnerability Index (SVI) [26], can be used to prioritize wastewater sites not only to fill clinical surveillance gaps but also to include vulnerable communities where such factors put people at greater risk of negative health outcomes [27]. This dual objective can then be achieved by investigating the spatial relationship between areas served by municipal wastewater systems and areas served by ED locations across the state, while also incorporating measures of social vulnerability to identify communities where expanded wastewater surveillance may provide the greatest public health benefit.

This study uses geographic information system (GIS) methods to identify candidate wastewater surveillance sites that could expand and strengthen North Carolina’s infectious disease surveillance infrastructure. Correlation analyses between CLI ED visit data and wastewater viral concentrations were initially conducted to evaluate alignment between the two surveillance systems. We then used GIS to define areas beyond a 20-minute travel time to an ED as low accessibility and used statewide sewershed polygons to identify sewersheds overlapping at least 50% with these low-accessibility areas. Sites where sewershed populations had moderate to high SVI scores were then prioritized for enrollment to expand surveillance reach to populations potentially underrepresented in existing ED and wastewater systems.

## Methods

### Wastewater and emergency department data correlation

A correlation analysis was conducted to assess alignment between wastewater data and emergency department (ED) visit data for SARS-CoV-2. Wastewater samples were collected twice weekly by participating utilities and transported to the North Carolina State Laboratory for Public Health (SLPH) and partner academic laboratories for analysis. Utility participation in the program is voluntary, and all sampling was performed by utility personnel at designated field sites; the study team did not conduct on-site sampling or any activities requiring permits. SARS-CoV-2 RNA concentrations in wastewater were obtained from 20 sampling sites within the North Carolina Wastewater Monitoring Network (NCWMN) whose sewersheds included at least one ED that consistently reported data from the week ending November 20, 2021, through the week ending August 3, 2024. Samples were analyzed using reverse transcriptase droplet digital polymerase chain reaction (RT-ddPCR) as previously described [23]. The 34-month study period was selected to capture multiple transmission peaks during and after the COVID-19 pandemic. For weeks in which more than one sample was collected at a site, population-normalized SARS-CoV-2 concentrations were averaged to produce a weekly value.

Within the NC DETECT system, ED visits from all 132 hospitals were classified into the COVID-like Illness (CLI) syndrome based on a keyword and diagnosis code-based search within the chief complaint, triage note, and diagnosis code fields for each visit. The CLI definition comprises of International Statistical Classification of Diseases and Related Health Problems, 10th Revision, Clinical Modification (ICD-10-CM) diagnosis codes (B97.2 or B34.2, J12.81 or J12.82, or U07.1 or U07.217) [28] or one of the following conditions: a chief complaint related to coronavirus, triage notes indicating a loss of sense of taste or smell, or triage notes indicating shortness of breath or respiratory distress and fever or chills. CLI visits with diagnosis codes for influenza (J09–J11.89) were excluded unless they had one of the ICD-10-CM inclusion codes [29]. The date for each CLI record was the ED visit date. ED visit percentages were calculated as a proportion of weekly ED visits for CLI divided by the total weekly ED visits, from the week ending November 20, 2021, through the week ending August 3, 2024.

Spearman’s rank-order correlation analysis was conducted to assess the association between SARS-CoV-2 RNA concentrations in wastewater in the sewershed and the corresponding ED visit percentages for CLI for each hospital. A non-parametric approach was used because the data was not normally distributed and was measured on two different scales. After importing the weekly time series data, rows with missing (NA) values were removed to ensure pairwise completeness, and data were visually inspected for outliers. A Spearman correlation coefficient (ρ) and p-value were calculated for each pairing of CLI and wastewater data and were considered statistically significant when p < 0.05. Correlation analyses were performed in R version 4.5.0 [30].

### Geospatial analysis of emergency department drive-time and sewershed areas

Drive-time areas to 133 civilian emergency departments (EDs) in North Carolina (including one hospital not currently reporting to NC DETECT) were calculated to identify areas with limited accessibility to ED services based on travel times by vehicles under typical travel conditions. We chose drive-times because they are a better measure of accessibility than Euclidean distance or buffer zones [31] and are therefore a good indicator of geographic disparity in healthcare access [32]. ED addresses were obtained from NC Division of Health Service Regulation data [33] and geocoded to produce input points for the drive-time tool. These geocoded point features were visually inspected with orthoimagery to ensure correct locations. We then created 20-, 30-, 40-, and 60-minute drive-time areas to each emergency department location, and the individual ED drive-time polygons were dissolved to create a single statewide drive-time area for each time step. Next, each drive-time area was clipped by the next lower drive-time area to produce drive-time polygons representing areas that were 0–20-, 20–30-, 30–40-, and 40–60-minute travel time to emergency departments by vehicle. Finally, the 20–30-, 30–40-, and 40–60-minute polygons were dissolved to create a polygon to represent low-accessibility areas or those which were 20–60 minutes away from EDs. All service areas were also clipped to the NC state boundary to reflect drive times only within the state. The drive time areas were created using Esri’s Network Analyst tools in ArcGIS Online. All additional geospatial processing was performed in Esri’s ArcGIS Pro v3.1 [34].

A statewide sewershed polygon dataset was created for North Carolina’s wastewater treatment plants (WWTPs). First, sewershed polygons for 51 WWTPs, previously collected by NC DHHS for sites participating in the NCWMN, were merged with Type A Current Public Sewer Systems (2004) polygons downloaded from NC OneMap [35]. If a sewershed polygon was present in both the NCWMN data and the NC OneMap data, the NCWMN sewershed polygon was retained. The sewershed polygons were visually inspected and small, disconnected areas of the sewershed were removed. Next, each sewersheds total population and social vulnerability was calculated. Sewershed populations were estimated from the Environmental Protection Agency’s Dasymetric Allocation of Population, 2020 raster data using zonal statistics [36]. The CDC’s Social Vulnerability Index (SVI) assigns percentile ranks to US Census tracts and counties based on 16 Census-derived variables related to social vulnerability, such as poverty, crowded housing, race, and disability [26]. In addition to an overall vulnerability score, SVI provides theme-specific indices for socioeconomic status, household characteristics, racial and ethnic minority status, and housing type and transportation, allowing assessment of whether specific dimensions of vulnerability are driving the overall SVI. We calculated the sewershed’s overall social vulnerability rank and ranks for each SVI theme. To do this, we selected census tracts that intersected the sewershed polygons and calculated the population-weighted average of SVI ranks.

We limited our analysis of potential sewersheds to those that were not actively sampling with NCWMN and served greater than 1200 people based on the population estimates calculated above. For optimum sensitivity, we chose a population cut-off that would include smaller sites but exclude those where data might need to be suppressed due to data privacy concerns. To identify wastewater sites that served areas with overall low access to an ED, sewershed polygons were intersected with the 20–60-minute drive-time polygon and the area and the percent overlap for each was calculated. The 20–30-, 30–40-, and 40–60-minute drive-time area polygons were also assessed to understand which drive-time category contained most of the overlap. Sites where the sewershed overlapped at least 50% with the 20–60-minute drive-time polygon and that had moderate-high (≥ 0.5) or high (≥ 0.75) overall SVI ranks were selected as new priority sites for enrollment in the NCWMN. We selected conservative criteria — at least 50% overlap and greater than 0.5 overall SVI — to create a more comprehensive list of potential sites in case other factors, such as a need for geographic coverage of a specific area or the wastewater utility’s ability to participate, might impact a site’s enrollment.

## Results

We identified 28 hospitals located within 18 NCWMN sewersheds that had consistent wastewater SARS-CoV-2 concentration data over the 34-month period; five wastewater sites served more than one hospital. Sites with consistent wastewater data submitted an average of two wastewater samples per week with minimal variation in sampling week to week (S1 Table). When emergency department (ED) and COVID-like illness (CLI) visit percentages were compared with wastewater SARS-CoV-2 concentrations, 26 hospitals demonstrated statistically significant (p < 0.05) moderate to strong correlation (rho = 0.303–0.718) with their corresponding sewershed data (S1 Table). The observed alignment between the two surveillance systems suggests that wastewater surveillance data complement clinical surveillance, particularly in communities where ED data may be lacking.

**Fig 1:**
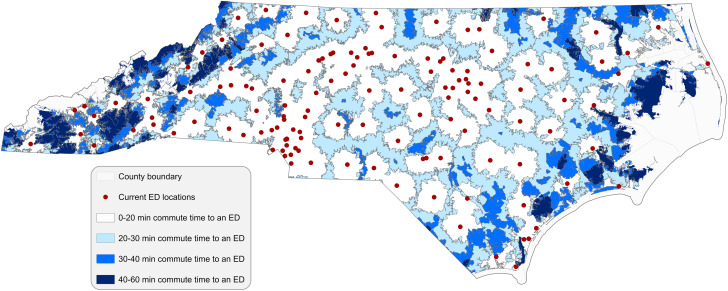
Map of North Carolina emergency department (ED) locations and drive-time polygons representing 0-20, 20-30, 30-40, and 40-60 minutes commute by car to an ED.

**Fig 2:**
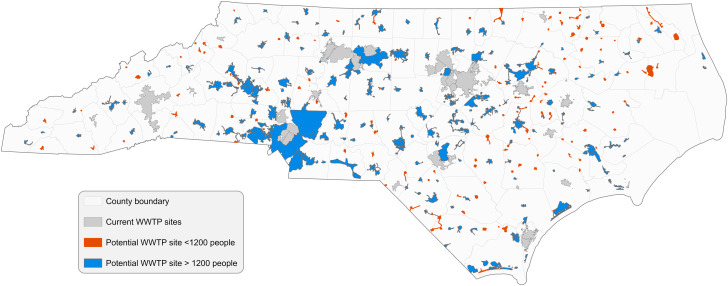
Map of North Carolina sewersheds symbolized by population. NC county boundaries are available from https://wastewaterw.nconemap.gov/.

Geospatial datasets depicting ED drive-time polygons (0–20, 20–30, 30–40, and 40–60 minutes) revealed that drive times to EDs were generally higher in the western and eastern regions of the state, where there were fewer EDs overall, and/or roadways traversed mountain slopes or coastal waterways (Fig 1). Out of 385 sewersheds statewide, 34 sewersheds currently monitored by NCWMN and 130 sewersheds serving populations under 1,200 were excluded, resulting in a final dataset of 220 sewersheds for further analysis. (Fig 2).

**Fig 3:**
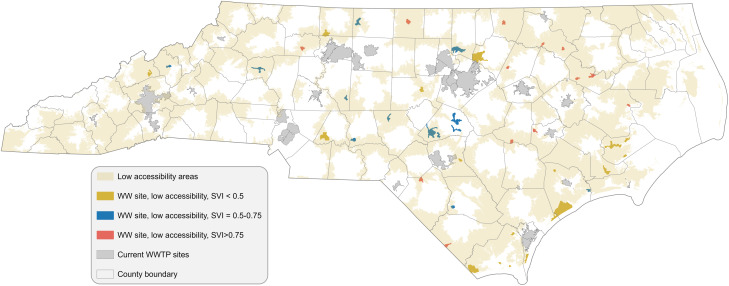
NC Wastewater Monitoring Network’s current sites (grey shading), and priority sites (> 1200 people and ≥ 50% overlap with 20–60-minute drive time; color coded by SVI scores), overlaid on areas with low accessibility to an ED (20–60-minute drive time areas). A site is represented as a sewershed on the map. NC county boundaries are available from https://wastewaterw.nconemap.gov/.

When we intersected the sewershed polygons with the 20–60-minute drive-time polygon, we identified 40 priority sites where at least 50% of the sewershed area overlapped with the ED low-accessibility area (Fig 3). These sites had relatively low populations (median = 2374) and represented 32 out of 100 North Carolina counties. Of the 40 sites with limited access to an ED, we found 26 sites with moderate to high Social Vulnerability Index (SVI) ranks, and 13 sites having high SVI ranks (Fig 3; Table 1). Sites with moderate to high SVI were located in 21 counties across the state and had a median population of 2010 people. High SVI sites were identified in 11 counties, mostly in the eastern part of the state, and had a median of 1900 residents (Table 1). The western part of the state included only one site, the Town of Burnsville, with low accessibility to an ED and moderatelyhigh overall SVI (0.663). Interestingly, of the 26 sites on the final priority list, the majority overlapped the most with the shortest drive-time polygon (20–30 minutes) (S2 Table). Only two sewersheds, Town of Belhaven and Town of Yanceyville, overlapped a high amount with the 30–40-minute drive-time polygon (79% and 71% respectively; S2 Table). Together, these sites represent priority candidates for enrollment in the NCWMN, as they would extend surveillance coverage to populations that may be underrepresented in ED clinical surveillance data.

**Table 1:** Overview of sewershed service areas including county, population, ED area percent overlap and SVI for wastewater treatment plants.

Wastewater Treatment Plant Name	County	Population	Overlap Percent	Overall SVI
Town of Warrenton	Warren	1315	100%	0.98
Town of La Grange	Lenoir	2494	58%	0.95
Town of Yanceyville	Caswell	1854	100%	0.94
Town of Red Springs	Robeson	3246	95%	0.93
Town of Belhaven	Beaufort	1391	90%	0.92
Town of Williamston	Martin	5127	60%	0.90
Town of Enfield	Halifax	1886	100%	0.89
Town of Mount Olive	Wayne	4132	100%	0.89
Town of Yadkinville	Yadkin	2969	100%	0.81
Town of Robersonville	Martin	1422	100%	0.79
Town of Spring Hope	Nash	1266	55%	0.79
Town of Scotland Neck	Halifax	1900	100%	0.77
Tabor City	Columbus	2312	100%	0.76
Town of Butner	Granville	5358	86%	0.68
Town of Burnsville	Yancey	1933	50%	0.66
Town of Madison	Rockingham	2087	100%	0.64
Town of Mayodan	Rockingham	2436	100%	0.63
Town of Bladenboro	Bladen	1692	50%	0.61
Town of Robbins	Moore	1233	100%	0.61
Harnett County	Harnett	21920	57%	0.60
Lyon Station SD	Granville	1579	60%	0.59
City of Creedmoor	Granville	4866	95%	0.56
Town of Mount Gilead	Montgomery	1206	69%	0.54
Town of Denton	Davidson	1326	100%	0.54
Town of Swansboro	Onslow	2088	100%	0.53
Town of Taylorsville	Alexander	3640	100%	0.51

## Discussion

The primary goal of this study was to identify new wastewater surveillance sites for potential enrollment in the North Carolina Wastewater Monitoring Network to fill gaps in clinical surveillance data and increase coverage of vulnerable communities. To justify our approach, we first examined the relationship between North Carolina’s NC DETECT and wastewater surveillance systems, which together provide complementary data on infectious disease trends at the community level. Specifically, we compared wastewater data from sampling sites with emergency department (ED) COVID-like illness (CLI) data from hospitals located within the corresponding sewersheds to assess alignment between the two systems. Correlations were assessed over an extended time to include data from the COVID-19 pandemic and post-pandemic periods to provide a more holistic overview of surveillance conditions. However, this may have led to weaker than expected correlations in some cases as the relationship between wastewater and clinical data can change due to variations in disease severity, population immunity, and healthcare seeking behavior over time. Nevertheless, considering the findings reported here and the positive correlations in previous studies, we believe that wastewater monitoring data can effectively serve as a proxy for communities where clinical surveillance data is limited or not collected [22,23].

Current NCWMN wastewater sampling sites are predominantly located within the 0–20-minute drive-time area, suggesting sampling of a primarily urban population that is also being captured by syndromic surveillance. Through this work, we were able to highlight 40 new wastewater sampling sites in semi-urban or rural areas where people live further away from an ED. We intentionally used a lower population cutoff to enhance the sensitivity of the analysis which enabled inclusion of 25 sites with populations less than CDC’s data suppression criteria that would have otherwise been missed [37]. We further refined our list of 40 sites to focus on populations with high social vulnerability. Measures of social vulnerability, such as lack of access to transportation, crowded living conditions, poverty, and no health insurance are linked to higher disease incidence and negative health outcomes [38,39]. By including these vulnerable populations in wastewater surveillance, we aimed to ensure people at higher risk for poor health are represented and to capture a more complete picture of the disease burden within communities across the state.

Thirteen sites in ED low-accessibility areas had an SVI rank higher than 0.75, indicating high to very high social vulnerability within these communities. After evaluating these sites further for individual SVI themes including socioeconomic status, household characteristics, racial and ethnic minority status, and housing type and transportation, we found that six of the highest-ranking sites were also among the top ten sites for each individual SVI theme. This suggests that the choice of sites based on overall social vulnerability ranking generally captured individual social vulnerability themes well. An evaluation of individual social vulnerability themes also provided additional insight into the factors driving the overall SVI ranking up in the 13 high SVI sites. For example, in the Town of Yadkinville, the SVI rank for housing type and transportation was 0.9, which was higher than the other SVI themes and the overall SVI, indicating that the community’s vulnerability is most related to lack of transportation or crowded living conditions (S2 Table). Communities in Tabor City and Scotland Neck have high socioeconomic status SVI rankings, indicating greater vulnerability related to poverty, unemployment, lack of health insurance, and lower educational attainment (S2 Table). These observations highlight the significance of socioeconomic factors, living conditions, and lack of transportation in potentially impacting people’s access to healthcare.

When prioritizing sites for enrollment, wastewater programs also need to weigh surveillance goals beyond increasing overall coverage and inclusion of vulnerable populations. For example, wastewater programs that find the greatest value in using wastewater data for emerging disease trends might prioritize sites that provide early warning [23,40]. Alternatively, to support targeted and timely outbreak responses, wastewater surveillance programs might prioritize sampling efforts at sites serving higher at-risk populations such as communities with low vaccination coverage or areas characterized by demographic factors associated with increased vulnerability. Finally, the willingness and ability of the wastewater utility and/or the local health department to participate in wastewater surveillance needs to be considered. Limited financial or personnel resources have been known to limit a site’s ability to participate regardless of the importance of monitoring in that location.

Our study was subject to several limitations. First, we only analyzed sewersheds with more than 1200 residents so the data generated at these sites could be added to public-facing dashboards without risk of disclosing sensitive information. However, this population cut-off, despite being more inclusive, likely limited the number and geographic spread of the sites we identified because less-populated sewersheds were mostly located in the western and eastern parts of North Carolina where there were longer ED drive-times. Second, our statewide sewershed dataset was created by combining sewershed polygons provided to the NCWMN and publicly available sewer service areas. Therefore, some communities connected to municipal wastewater systems may have accidentally been left out of our analysis. Third, sewershed populations are not static; people who do not reside within a sewershed may travel into or out of it for school, work, or leisure. Consequently, the population contributing to wastewater may be difficult to determine, which may complicate comparisons between wastewater and clinical data. Finally, gaps in clinical surveillance data cannot always be filled by wastewater surveillance data. This is especially relevant in North Carolina where roughly 50% of the population utilizes septic and are thus not likely to be included in wastewater surveillance data [41]. In North Carolina, wastewater sampling from pooled septic systems and pump stations upstream of small to moderately sized neighborhoods has shown some success in monitoring for COVID-19 [42]. Given sufficient funding, this approach could be employed to enhance wastewater surveillance coverage of small, rural populations.

## Conclusions

The North Carolina Department of Health and Human Services employs multiple surveillance systems, including wastewater and clinical data streams, to monitor infectious disease trends across the state. This analysis demonstrates the complementary strengths of wastewater surveillance and emergency department (ED) visit data in tracking COVID-19 activity and identifies opportunities to strengthen public health surveillance using wastewater data. It also provides a framework for expanding wastewater surveillance in regions with limited ED accessibility (20–60-minute drive time to an ED) and high social vulnerability. In states like North Carolina with substantial rural populations, this novel strategy helps efficiently address surveillance gaps and reduce disparities while making strategic use of limited public health resources for selecting wastewater sampling sites. This, in turn, can help enhance public health preparedness by enabling the detection of emerging threats in populations that are often underrepresented in traditional surveillance systems.

## Supporting information

S1 Table Spearman’s correlation coefficient (rho) for wastewater treatment plant (WWTP) sites comparing average, normalized concentration measurements for SARS-CoV-2 in wastewater to emergency department visit rates for COVID-19-like illness. (GIF)

S2 Table Wastewater sampling sites that overlapped at least 50% with the 20–60-minute drivetime to an ED and had high SVI > 0.50. (TIFF)
